# Comparison of libido, Female Sexual Function Index, and Arizona scores in women who underwent laparoscopic or conventional abdominal hysterectomy

**DOI:** 10.4274/tjod.78803

**Published:** 2017-06-15

**Authors:** Semra Kayataş, Enis Özkaya, Murat Api, Seyhan Çıkman, Ayşen Gürbüz, Ahmet Eser

**Affiliations:** 1 Zeynep Kamil Women and Children’s Health Training and Research Hospital, Clinic of Obstetrics and Gynecology, İstanbul, Turkey

**Keywords:** sexual function, libido, hysterectomy, Female Sexual Function Index, Arizona Sexual Experiences Scale

## Abstract

**Objective:**

The aim of the present study was to compare female sexual function between women who underwent conventional abdominal or laparoscopic hysterectomy.

**Materials and Methods:**

Seventy-seven women who were scheduled to undergo hysterectomy without oophorectomy for benign gynecologic conditions were included in the study. The women were assigned to laparoscopic or open abdominal hysterectomy according to the surgeons preference. Women with endometriosis and symptomatic prolapsus were excluded. Female sexual function scores were obtained before and six months after the operation from each participant by using validated questionnaires.

**Results:**

Pre- and postoperative scores of three different quationnaires were found as comparable in the group that underwent laparoscopic hysterectomy (p>0.05). Scores were also found as comparable in the group that underwent laparotomic hysterectomy (p>0.05). Pre- and postoperative values were compared between the two groups and revealed similar results with regard to all three scores (p>0.05).

**Conclusion:**

Our data showed comparable pre- and the postoperative scores for the two different hysterectomy techniques. The two groups were also found to have similar pre- and postoperative score values.

## PRECIS:

Comparable pre- and the postoperative sexual function scores were obtained following two different hysterectomy techniques.

## INTRODUCTION

Hysterectomy is a commonly performed surgical procedure^(1)^. Hysterectomy can be performed via the vaginal route or by applying minimally invasive techniques (laparoscopy, robotic surgery). Approximately 50% of cases undergo concomitant bilateral oophorectomy^(2)^, as a consequence, estrogen deficiency may influence women’s health. Furthermore, estrogen and androgen deficiency secondary to oophorectomy may aggrevate climacteric symptoms and sexual dysfunction, which may affect sexual pleasure, comfort, and frequency, resulting in lower desire, arousal, lubrication and sexual satisfaction. In addition, coital pain is a frequent sexual problem reported after perimenopausal oophorectomy^(3)^.

Some validated and non-validated quastionnaires introduced the term “sexual function” as an overall descriptive term for outcomes^(4)^ that include sexual activity and sexual function in terms of specific functional aspects, as well as satisfaction with sexual activity.

Age, menopausal status, systemic diseases, and also gynaecologic surgery were reported to adversely affect the sexual response^(5)^. It was reported that gynaecologic surgery may interfere with sexuality in middle-age women and some factors played a significant role leading to dysfunction, including changing self-image, sexual pain and orgasm difficulty^(6)^. Sexual wellbeing may differ according to the type of hysterectomy because different techniques damage the innervation and supportive structures of the pelvic floor. Recent technical advances made laparoscopic surgery possible in many surgical fields and laparoscopy became a feasable technique for hysterectomy^(7)^.

In this study, we aimed to assess the effect of laparotomic versus laparoscopic hysterectomy techniques on the Female Sexual Function Index (FSFI), the Libido Scoring System (LSS), and Arizona scores.

## MATERIALS AND METHODS

### Participants

This prospective observational study was performed at the Gynecology Clinic of Zeynep Kamil Women and Children’s Health Training and Reseach Hospital between June 2014 and December 2015. Informed consent was obtained from each participant.

Seventy-seven consecutive women who were sexually active and healthy premenopausal patients, aged between 40-55 years, and were offered hysterectomy for benign indications either via laparotomy or laparoscopy were included in the study. The technique of hysterectomy was determined according to the surgeon’s preference. Exclusion criteria consisted of endometriosis, symptomatic prolapsus, chronic pelvic pain and malignancy as indications for surgery, patients with sexual dysfunction, participation refusal or reduced capability of understanding the survey, patients with severe depression or had been using antidepressant treatment, and patients whose partner had a severe illness or had died recently. Patients who required oophorectomy during the operation (n=3), developed complications in the postoperative period (n=2), and those who refused to participate in the study after the operation (n=6) were excluded.

The FSFI, Arizona, and LSS questionnaires were completed in face-to-face sessions. Sociodemographic data (personal and partner) were recorded. In total, 66 patients completed these questionnaires for evaluating sexual function prior to and six months after hysterectomy. Hysterectomies were performed by the same surgical team according to standard surgical techniques.

### The Female Sexual Function Index

The FSFI is a validated self-administered questionnaire that consists of 19 questions and measures six domains of sexual function: desire, arousal, lubrication, orgasm, satisfaction and pain^(8)^. The first and second questions have scales from 1 to 5 and other questions have scales from 0 to 5 for scoring. Scores obtained in a particular domain are added and multiplied by a respective factor (coefficients for questions 1-2: 0.6; 3-10: 0.3; 11-19: 0.4), which homogenizes the influence of each dimension. A total sum of each score is obtained and higher scores indicate healthy sexual life. Score ranges between 1.2 and 36. An optimal cut-off was introduced as 26^(9)^. In the present study, an FSFI score of 26 or less was defined as sexual dysfunction. Validation of this questionnaire in Turkish population was shown in previous reports^(10)^.

### The Arizona Sexual Experiences Scale

The Arizona Sexual Experiences Scale (ASEX) has five items that assess sexual experiences including: drive, arousal, vaginal lubrication, ability to reach orgasm, and satisfaction with orgasm. The lubrication item is assessed by the versions specific for sex. Each item is rated with a six-point scale. Scores range between 5 and 30; higher scores indicate better sexual life. Use of this questionnaire was shown in previous reports^(11)^.

### The Libido Scoring System

LSS was developed in 1997 by Api et al.^(12)^. It comprises four questions on four domains: orgasmic function, coital frequency, sexual desire, and sexual self-interest (masturbation). FSFI was well-correlated with the LSS, revealing a correlation coefficient of 0.96 (p<0.001) (the Cronbach’s α coefficient was found as 0.83 and the total kappa values were 0.67 and 0.77)^(12)^. The patients were scored and a total score less than 3 was considered as loss of libido; scores of 3 and 4 were considered as low libido, 5-7 as moderate libido, and 8-12 as high libido. The researcher asked the following questions to the patients:

1. How often do you have sex or masturbate?

2. Do you masturbate?

3. Who starts the sexual activity? (Who asks for or implies sex first?)

4. Do you have orgasm during masturbation or sexual intercourse?

### Statistical Analysis

Data were analyzed using SPSS 15.0 for Windows. Student’s t-test was used to compare continuous variables between the groups. The paired samples t-test was used to show comparisons of continuous variables before and after intervention. P values <0.05 were accepted as statistically significant.

## RESULTS

The groups were similar with regard to their mean age (44 vs. 46 years) and mean uterine volume (730 vs. 1050 cm3, p>0.05). The pre- and postoperative scores of the three different quationnaires were found as comparable in the group that underwent laparoscopic hysterectomy (p>0.05, Table 1). Scores were also found as comparable in the group that underwent laparotomic hysterectomy (p>0.05, Table 2). Pre- and postoperative values were compared between the two groups, which revealed similar results with regards to all three scores (p>0.05, Table 3).

## DISCUSSION

In this study, we compared the pre- and the postoperative sexual function of women who underwent laparoscopic or laparotomic hysterectomy. Our data revealed comparable pre- and postoperative scores between the groups. The comparison of pre- and postoperative score values within each group revealed similar results. We preferred to assess sexual function at the 6^th^ postoperative month based on a study that indicated a requirement of 6 months for pelvic innervation recovery^(13)^.

Hysterectomy is a frequently performed gynecologic surgery for variable indications. Sexual function after hysterectomy with different techniques has been questioned in several trials and generally it was thought that injury to the uterovaginal plexus during hysterectomy might interfere with the neuronal support of vagina, which leads to affected orgasm and lubrication^(14,15)^. There are also some data indicating similar sexual function in women with and without cervical ablation^(16,17)^, and studies also showed similar sexual function among different hysterectomy techniques including total abdominal hysterectomy, subtotal hysterectomy, and vaginal hysterectomy^(18)^.

A recently published review on this issue assessed the results of previously published studies and summarized the results under different headings including sexual desire, sexual arousal, orgasm, dyspareunia and sexual satisfaction, and the authors reported contradictory results with regard to these sexual parameters^(19)^. All these parameters were assessed by using three different questionnaires in our study. We found similar pre- and postoperative scores for each technique.

In contrast to our results, some studies in the literature showed measurable advances in life style and sexual function after simple hysterectomy, whereas others revealed negative results^(20,21,22,23)^. Furthermore, a previous study indicated that hysterectomy contributed to quality of life with minimal postoperative morbidity after minimally invasive surgery^(24)^.

Consistent with our results, according to some prospective, randomized studies, late psychosexual changes have not been thought to primarily depend on the surgical method (vaginal, abdominal total or subtotal hysterectomy); studies showed the quality of sexual relationship before the operation as the most significant predictive factor^(25)^.

The aforementioned conclusions show several conflicting results with regard to the impact of hysterectomy on sexual function. Some authors suggested that the parameters of questionnaires were unsatisfactory for assessing sexual function^(26)^.

Meta-analyses on this issue revealed that prolapsus operations, particularly posterior repairs using levator plication, seemed to deteriorate sexual function, and hysterectomy was found to improve sexual function, regardless of whether it was subtotal or total. The review concluded that gynecologic operations might influence sexual function; however, little validated data are available to come to this conclusion^(27)^. Due to the lack of validated data, we performed this study by using three different scoring systems including ASEX, which was shown to be a valid and reliable instrument for use in clinical trials on sexual function in the Turkish population^(28)^, and FSFI scoring, which has been used in several different studies in the Turkish population; validation of this scoring system for the Turkish population has been shown^(29,30)^. Finally, we also tried to confirm our results by the third different scoring system introduced by the Api et al.^(12)^ who concluded that this simple test provided a reliable measure for routine clinical practice or trial purposes.

Most of the studies in the literature included heterogeneous groups of participants to assess sexual function and the majority failed to exclude patients with certain factors (e.g., menopausal status, comorbidity, oophorectomy, endometriosis, malignity), which may interfere with the results^(31)^. Our data originated from a homogeneous group of patients from a single tertiary referral center; most women with the aforementioned factors that may be interfere with the results were excluded from our study and we used three different questionnaires comprising different parameters to assess the effect of surgical technique on sexual function.

### Study Limitations

Small sample size is the major drawback in our study.

## CONCLUSION

Our data showed comparable pre- and the postoperative scores for the two different hysterectomy techniques. Pre- and postoperative scores were similar within each surgical technique, using three different questionnaires revealed no effect of surgical technique on sexual function after hysterectomy.

## Figures and Tables

**Table 1:**
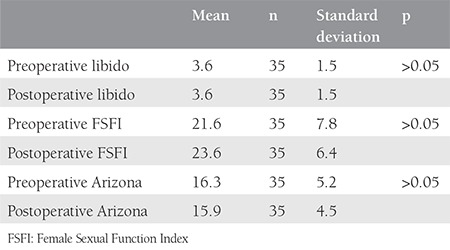
Comparison of pre- and postoperative scores of three different sexual function evaluation questionnaires in women who underwent laparoscopic hysterectomy

**Table 2:**
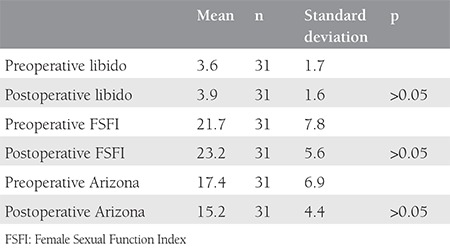
Comparison of pre- and postoperative scores of three different sexual function evaluation questionnaires in women who underwent laparotomic hysterectomy

**Table 3:**
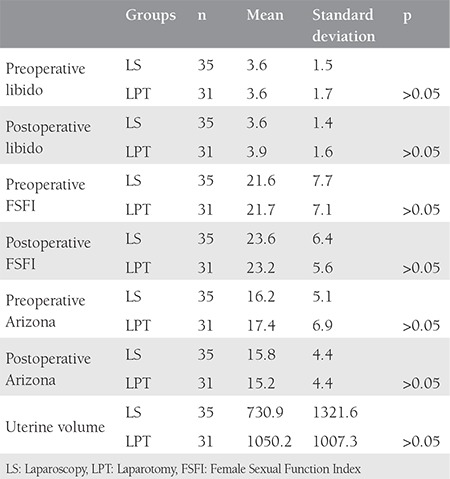
Comparison of three different sexual function evaluation questionnaire scores between women underwent laparoscopic and laparotomic hysterectomy
